# ASSESSING THE UPTAKE OF CORE OUTCOME SETS IN RANDOMIZED CONTROLLED TRIALS FOR PARKINSON’S DISEASE

**DOI:** 10.1093/geroni/igad104.3183

**Published:** 2023-12-21

**Authors:** Joanna George, Eli Paul, Shaelyn Ward, Kyle Fitzgerald, Garrett Jones, Kimberly Magana, Alicia Ford, Matt Vassar

**Affiliations:** Oklahoma State University College of Osteopathic Medicine, Tulsa, Oklahoma, United States; Oklahoma State University, Tulsa, Oklahoma, United States; Oklahoma State University College of Osteopathic Medicine, Tulsa, Oklahoma, United States; Oklahoma State University College of Osteopathic Medicine, Tulsa, Oklahoma, United States; Oklahoma State University College of Osteopathic Medicine, Tulsa, Oklahoma, United States; Oklahoma State University College of Osteopathic Medicine, Tulsa, Oklahoma, United States; Oklahoma State University Center for Health Sciences, Tulsa, Oklahoma, United States; Oklahoma State University Center for Health Sciences, Tulsa, Oklahoma, United States

## Abstract

**Background:**

Parkinson’s Disease (PD) affects 1% of adults aged 60 and older, including 10 million individuals worldwide. As PD’s incidence rises, research funding is increasing substantially. PD’s core outcome set (COS) provides standardization for PD clinical trial outcomes. Adherence to the PD COS may improve research quality and study comparability. Objective: Analysis of the COS uptake rate before and after the PD COS publication.

**Methods:**

On June 26th, 2023, we conducted a search of ClinicalTrials.gov to retrieve phase III/IV adult PD trials between 2013 and 2023. Screening for inclusion and data extraction occurred in a masked, duplicate fashion. Trial characteristics and COS uptake rate were extracted from this sample.

**Results:**

In our 111 included trials, the COS uptake rate was highest for the ‘Walking and Balance’ outcome and lowest for the ‘Hospital Admissions’ outcome. Overall, there was a non-significant monthly increase of 0.26% (P = 0.266, CI = [-0.20, 0.72]) in “COS-defined outcome” measurement when comparing pre- and post-COS publication.

**Conclusion:**

Overall, our study found no significant increase in COS uptake in PD clinical trials. We found multiple outcomes to be vastly unmeasured and heterogeneity among the measurement instruments used. These findings introduce complexity to the standardization and comparability of RCT outcomes. Barriers to COS uptake may be complex; however, overcoming these factors is vital to improving the usefulness of PD research.

